# Incidence of Research Gap Years in Orthopaedic Residency Applicants: The New Standard?

**DOI:** 10.5435/JAAOSGlobal-D-21-00247

**Published:** 2021-11-15

**Authors:** Eric J. Cotter, Evan M. Polce, Eric Lee, Kathryn L. Williams, Andrea M. Spiker, Brian F. Grogan, Gerald J. Lang

**Affiliations:** From the Department of Orthopedics and Rehabilitation, University of Wisconsin School of Medicine and Public Health, Madison, WI (Dr. Cotter, Mr. Polce, Dr. Lee, Dr. Williams, Dr. Spiker, Dr. Grogan and Dr. Lang).

## Abstract

**Introduction:**

The purpose of this study was to (1) determine the incidence of a research gap year (RGY) in orthopaedic residency applicants at a single institution over a seven-year span; (2) compare applicant characteristics between applicants who did a RGY with those who did not, and (3) report variables associated with match success for RGY applicants.

**Methods:**

Applicants who reported taking a year out from medical school to pursue research on their Electronic Residency Application Service to a single institution from 2014 to 2015 through 2020 to 2021 were reviewed.

**Results:**

A strong positive correlation was noted between the percentage of applicants who participated in a RGY and time (Pearson correlation: *r* = 0.945 [95% confidence interval (CI), 0.666­0.992], *P* = 0.001). Over the study period, 11% of applicants had done a RGY, most commonly after their third year of medical school (82.7%). Most RGY applicants matched orthopaedics (72.8%) and 19.4% matched at the same institution they did their RGY.

**Conclusion:**

The percentage of RGY applicants to the study institution nearly doubled between 2014 to 2015 and 2020 to 2021. RGY applicants had a higher match rate than nationally published match rates. Further study is needed on a national level.

The number of applicants applying to orthopaedic surgery residency programs continues to increase annually.^1-3^ Several applicant characteristics have been correlated with greater match success, including higher US Medical Licensing Examination (USMLE) step 1 scores,^4-6^ admission into the Alpha Omega Alpha (AOA) honor society,^5,6^ and having a greater mean number of applicant self-reported research activities in matched applicants (4.6 vs 3.0 from applicant data 2007 to 2014).^5^ USMLE Step 1 will become pass/fail in 2022, leading to speculation on what applicant factors will increase in importance as a result. A recent survey study of orthopaedic residency program directors aimed at answering this question. The results noted that USMLE Step clinical knowledge (CK) will become much more important; however, published research experiences were also noted to be a variable that will likely increase in importance.^7^

Our experience has been that a greater percentage of recent orthopaedic surgery residency applicants are taking a research gap year (RGY) at some point during or following medical school. To date, limited data exist within orthopaedics and medical education as a whole regarding the incidence of RGYs and the effect they may have on match success of applicants in a desired specialty. A survey study of integrated plastic surgery residency applicants over a four-year span reported 25% of all applicants had participated in a RGY. The authors noted that 63% of RGY applicants stated they pursued the RGY to strengthen their residency application.^8^ A similar study in radiation oncology residency applicants found 33% of applicants did a RGY. Within orthopaedics, the incidence of applicants seeking RGYs, the reasons students are seeking these opportunities, and whether participating in a RGY leads to higher match success into orthopaedic surgery residency are unknown. The lone orthopaedic study investigating this topic to date was a review of a single academic institution's 18-year experience with offering RGY opportunities, noting a higher match rate in these students despite a four-point lower average step 1 score than publicly published national data.^2^

The purpose of this study was to: (1) determine the incidence of RGY in residency applicants to a single academic institution over the past seven application cycles; (2) compare applicant characteristics between students who did a RGY with those who did not from a single academic institution's applicant pool over seven years, and (3) report variables associated with match success for applicants who did a RGY. It was hypothesized that there would be an increasing percentage of orthopaedic residency applicants to this institution who did a RGY during or immediately after medical school over time. In addition, it was hypothesized that applicants who did a RGY would, on average, have lower USMLE Step 1 scores and lower AOA percentage than applicants who did not do a RGY.

## Methods

**Figure 1:**
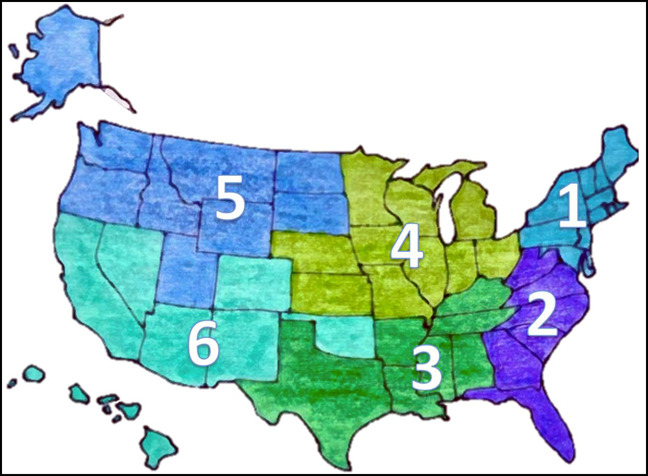
Map of the United States color coded into geographic regions for analysis purposes. Washington DC was considered part of region 2. Regions outside of the United States were collectively considered “international” (region 7).

This study was granted exemption by the IRB. Demographic, medical school location, USMLE step scores, AOA status, research productivity (presentations, publications, book chapters, and grants), and whether an applicant took a RGY or not was reviewed for all medical student applicants to a single academic institution's orthopaedic surgery residency program from the 2014 to 2015 application cycle through the 2020 to 2021 application cycle. All podium and poster presentations, book chapters, abstracts, and publications (including online publications and publications listed on an ERAS application that were under review) were aggregated for each applicant to obtain total research activities. By combining all discrete research activities, a comprehensive picture of research productivity could be obtained for each applicant. Geographic locations were determined by Figure 1.

### Research Gap Year Applicant Information

Medical students who did a full academic year of dedicated research were identified from their responses to the ERAS application prompt regarding any interruptions in medical school training. Applicants who clearly stated they took a year out from medical school for a Master of Public Health (MPH), Master of Business Administration (MBA), or formal PhD program or did less than nine months of a dedicated research fellowship were not considered RGY applicants for the purposes of this study. In addition to the aforementioned data variables extracted from each application, several variables were recorded for students identified as having done a RGY. Specifically, the location of where they did a RGY was determined either by their explanation for why they took a year off from medical school on their ERAS application or by searching PubMed for any peer-reviewed publications and comparing the listed author affiliation to that of their medical school listed on their ERAS application. Although an indirect link, it was presumed if an applicant was a co-author on several publications at a medical center that was distinct from their reported medical school on ERAS, that this was likely the location of where they were for their RGY. In addition, the applicant was searched in Google (Alphabet) to see if they ultimately matched successfully into an orthopaedic residency program or not. A combination of program websites, professional social networking sites, and published abstracts were used through Google search to try and identify applicants. More than 70% of applicants were able to be successfully identified by this method using their reported medical school and year of application to residency listed on ERAS as a cross-reference to confirm accurate identification with what was seen online. This search method could not be used for the most recent application cycle, 2020 to 2021, because most orthopaedic programs have not yet updated residency websites to reflect match results.

### Statistical Analysis

Shapiro-Wilk testing was done to identify whether variables were normally distributed. Based on whether the assumption of normality was met, descriptive statistics including mean and standard deviations or median and interquartile ranges were calculated for continuous variables. Counts and percentages were reported for categorical data. Nonparametric Wilcoxon rank-sum tests or parametric Student two-sample *t*-tests were used to compare continuous data between RGY applicants who matched and those who did not match and between RGY and non-RGY applicants. The Pearson product-moment correlation coefficient was calculated to determine the association between the percentage of RGY applicants and time. Categorical data were evaluated with chi-squared tests. Receiver operating characteristic (ROC) curve with area under the ROC curve (AUROC) analysis was done using the cutpointr package to determine the quantity of research output during a RGY associated with a greater likelihood of matching into orthopaedic surgery residency.^9^ The optimal research output cutoff threshold was calculated through maximizing sensitivity and specificity with Youden index^10^ Generally, AUROC values of 0.5 to 0.69 are considered poor, 0.7 to 0.8 acceptable, 0.8 to 0.9 excellent, and greater than 0.9 outstanding^11^ *P <* 0.05 was considered statistically significant. All statistical analyses were done using RStudio software version 4.0.4 (R Foundation for Statistical Computing).

## Results

### Research Gap Year Applicants

Of 5,095 total applicants to the study institution from 2014 to 15 to 2020 to 21, 558 (11%) were RGY applicants. Most applicants pursued a RGY after their third year of medical school (82.7%), with 9.8% pursuing a RGY after their fourth year of medical school. The timing of when applicants took a RGY remained relatively constant during the study period. The median number of research activities for the RGY applicant cohort at the time of application submission was 21. No significant differences were identified between median total lines on curriculum vitae based on timing of RGY (*P* > 0.05 for all). For the after M1 year cohort, the median number of research activities was 15 (IQR 12 to 19); after M2 year, 18 (IQR 10 to 32); after M3 year, 22 (IQR 14 to 36); and after M4 year, 18 (IQR 12 to 34). A complete description of applicant demographics, median USMLE Step 1 scores, timing of RGY, medical school location, and research productivity at time of residency application for each application cycle and in total are summarized in Table 1.

**Table 1:** General Characteristics of the RGY Cohort Regarding Application Cycle Year

	2014-2015	2015-2016	2016-2017	2017-2018	2018-2019	2019-2020	2020-2021	Total
Number of RGY applicants (%)^a^	50 (7.4)	67 (9.3)	80 (10.8)	84 (11.3)	76 (11.1)	100 (13.3)	101 (13.0)	558 (11.0)
Female sex (%)	6 (12.0)	12 (17.9)	11 (13.8)	18 (21.4)	18 (23.7)	18 (18.0)	19 (18.8)	102 (18.3)
USMLE step 1 score	241 (226-250)	242 (228-250)	243 (231-253)	241 (229-250)	243 (233-248)	242 (232-251)	242 (231-253)	242 (230-251)
AOA (%)	5 (10.0)	11 (16.4)	16 (20.0)	19 (22.6)	11 (14.5)	11 (11.0)	18 (17.8)	91 (16.3)
Total lines on CV	12 (7-23)	15 (12-26)	18 (11-29)	22 (15-31)	22 (15-44)	26 (16-36)	26 (18-47)	21 (13-36)
Timing of RGY (%)^b^								
Before M1	0 (0.0)	0 (0.0)	0 (0.0)	1 (1.4)	0 (0.0)	0 (0.0)	0 (0.0)	1 (0.2)
After M1	0 (0.0)	0 (0.0)	0 (0.0)	2 (2.7)	0 (0.0)	0 (0.0)	0 (0.0)	2 (0.4)
After M2	2 (4.1)	4 (6.2)	9 (11.3)	4 (5.4)	6 (10.7)	2 (2.5)	7 (7.4)	34 (6.8)
After M3	40 (80.0)	50 (76.9)	64 (80.0)	59 (79.7)	48 (85.7)	70 (88.6)	81 (85.3)	412 (82.7)
After M4	7 (14.3)	11 (16.9)	7 (8.8)	8 (10.8)	2 (3.6)	7 (8.9)	7 (7.4)	49 (9.8)
Medical school location (%)								
Northeast	17 (34.0)	17 (25.4)	29 (36.3)	25 (29.8)	21 (27.6)	37 (37.0)	36 (35.6)	182 (32.6)
Southeast	4 (8.0)	6 (9.0)	12 (15.0)	14 (16.7)	15 (19.7)	17 (17.0)	12 (11.9)	80 (16.1)
South	3 (6.0)	4 (6.0)	3 (3.8)	4 (4.8)	2 (2.6)	4 (4.0)	9 (8.9)	28 (5.6)
Midwest	13 (26.0)	24 (35.8)	19 (23.8)	26 (31.0)	21 (27.6)	30 (30.0)	27 (26.7)	160 (32.1)
Northwest	1 (2.0)	2 (3.0)	1 (1.3)	0 (0.0)	1 (1.3)	0 (0.0)	2 (2.0)	7 (1.3)
Southwest	11 (22.0)	12 (17.9)	13 (16.3)	8 (9.5)	15 (19.7)	10 (10.0)	12 (11.9)	81 (16.3)
International	2 (4.0)	2 (3.0)	3 (3.8)	7 (8.3)	0 (0.0)	2 (2.0)	3 (3.0)	19 (3.8)

AOA = alpha omega alpha medical honor society, IQR = interquartile range, RGY = research gap year, USMLE = US Medical Licensing Examination

a Percentage represents the number of RGY applicants divided by the total number of applicants from each respective cycle

b Data on RGY timing were available for 498 (89.2%) applicants. Percentages are relative to the number of applicants with available data in each respective cycle.

Continuous data presented as median (IQR) unless specified otherwise.

**Figure 2:**
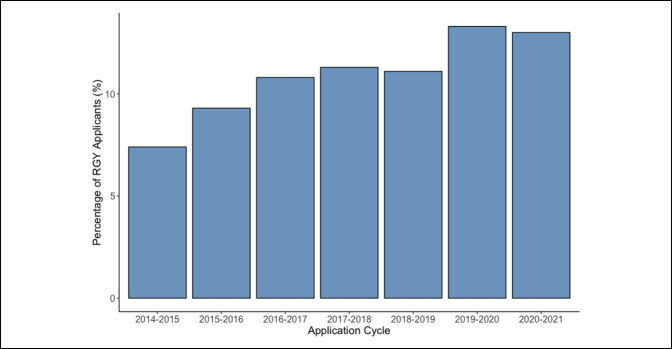
Histogram demonstrating the percentage of research gap year (RGY) applicants relative to the total number of applicants with respect to each application cycle.

A strong positive correlation was found between the percentage of applicants to the study institution who participated in a RGY and time (Pearson correlation: *r* = 0.945 [95% CI, 0.666-0.992], *P* = 0.001). The percentage of RGY applicants to the study institution nearly doubled between 2014 to 2015 and 2020 to 2021 (Figure 2).

### Geographic Location of Research Gap Year Programs

Overall, most RGY applicants to the study institution attended medical school in the Northeast (N = 182, 32.6%) or Midwest (N = 160, 32.1%). Similarly, the most common geographic locations to do a RGY for applicants applying to the study institution were the Northeast (N = 204, 38.7%) and the Midwest (N = 168, 31.9%). RGY applicants to the study institution most commonly matched into residency programs located in the Northeast (108, 33.1%) or Midwest (N = 99, 30.4%). See Table 2 for complete geographic information for RGY applicants to the study institution. Please see Appendix 1, http://links.lww.com/JG9/A168**,** for a complete breakdown of specific institutions where students did RGYs.

**Table 2:** Geographic Location of Medical School, RGY Institution, and Orthopaedic Residency Program for the RGY Cohort

Geographic Location	Medical School	RGY Institution	Orthopaedic Residency Program
Northeast	182 (32.6)	204 (38.7)	108 (33.1)
Southeast	80 (16.1)	35 (6.6)	41 (12.6)
South	28 (5.6)	23 (4.4)	18 (5.5)
Midwest	160 (32.1)	168 (31.9)	99 (30.4)
Northwest	7 (1.3)	7 (1.3)	12 (3.7)
Southwest	81 (16.3)	86 (16.3)	46 (14.1)
International	19 (3.8)	4 (0.8)	2 (0.6)

RGY = research gap year

Geographic data for medical school and RGY location were available for 557 of 558 (99.8%) and 527 of 558 (94.4%) of RGY applicants, respectively. Match data were not available for the 2020 to 2021 cycle. Of the remaining cycles analyzed, geographic data for matched orthopaedic residency program location were available for 326 of 457 (71.3%) applicants.

Data are displayed as count (percentage).

Most applicants who pursued a RGY matched into an orthopaedic surgery residency program (N = 330, 72.8%). RGY applicants who successfully matched had significantly higher median USMLE Step 1 scores (243 vs 236 for unmatched, *P* < 0.001), were significantly more likely to be AOA (20.3% vs 4.9% for unmatched, *P* < 0.001), and had significantly more research activities (mean 21 vs 16 for unmatched, *P* < 0.001). No differences were found in match success based on when applicants did a RGY (*P* > 0.05 for all). Table 3 details important application variables between RGY applicants who matched orthopaedic surgery compared with RGY applicants who did not. Match data were not available for the 2020 to 2021 cycle. Of the remaining cycles analyzed, data on matching at the same institution where applicants performed their RGY were available for 371 of 457 (81.2%) applicants. Seventy-two (19.4%) RGY applicants matched at the same institution where they did their RGY.

**Table 3:** Applicant Variables for Research Gap Year Applicants Who Matched Orthopaedics Compared With Research Gap Year Applicants Who Did Not Match Orthopaedics

	Matched Orthopaedics	Did Not Match Orthopaedics	*P*
Number (%)^a^	330 (72.8)	123 (27.2)	—
USMLE step 1 score	243 (232-252)	236 (222-245)	<0.001
USMLE step 2 CK score	250 (241-259)	241 (229-252)	<0.001
AOA (%)	67 (20.3)	6 (4.9)	<0.001
Total research activities	21 (14-37)	16 (9-28)	<0.001
Timing of RGY (%)^b^			
Before M1	1 (0.3)	0 (0.0)	> 0.999
After M1	1 (0.3)	1 (1.0)	0.457
After M2	17 (5.8)	9 (8.6)	0.635
After M3	246 (83.4)	84 (80.0)	0.525
After M4	30 (10.2)	11 (10.5)	> 0.999
Timing of RGY comparison¥			
After M2 vs after M3	M2: 17/26 (65.4%) matched	M3: 246/330 (74.5%) matched	0.428
After M2 vs after M4	M2: 17/26 (65.4%) matched	M4: 30/41 (73.2%) matched	0.686
After M3 vs after M4	M3: 246/330 (74.5%) matched	M4: 30/41 (73.2%) matched	> 0.999

AOA = alpha omega alpha medical honor society, CK = clinical knowledge, IQR = interquartile range, RGY = research gap year, USMLE = US Medical Licensing Examination

a Match data were not available for the 2020 to 2021 cycle. Of the remaining cycles analyzed, data on orthopaedic surgery match success were available for 453/457 (99.1%) applicants. Data were analyzed only for those applicants with available data on where they matched.

b Data on RGY timing were available for 404 (88.4%) applicants.

¥The before M1 and after M1 cohorts were not statistically compared with the other cohorts due to small sample sizes (N < 5)

Continuous data presented as median (IQR) unless specified otherwise.

Bold values indicate statistical significance (*P* < 0.05).

### Match Success Based on Research Output for Research Gap Year Applicants

**Figure 3:**
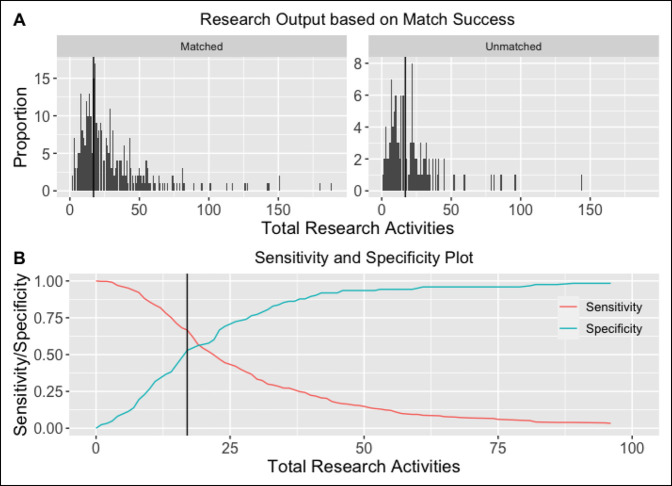
Association between research gap year (RGY) research output relative to orthopaedic surgery match success. **A,** Graph showing distribution of total research output (i.e., total discrete research activities listed on applicants' curriculum vitae [CV]) for RGY applicants regarding orthopaedic surgery match success: 330 RGY applicants ultimately matched into orthopaedic surgery, whereas 123 did not. The two vertical black lines correspond to the calculated research output threshold associated with greater match success (≥17 total research activities). **B,** Plot displaying the threshold sensitivities and specificities when the research output cutoff varies from 0 to 100 total research activities. The vertical black line represents the optimal research output threshold associated with a greater likelihood of matching; however, this threshold was ultimately found to be only poorly associated with match success based on ROC/AUC analysis. AUC = area under the curve; ROC = receiver operating characteristic

A poor association was found between total research activities and successful match into orthopaedic surgery for RGY applicants (receiver operating characteristic [ROC] curve cutoff threshold: ≥ 17 total research activities; area under the ROC curve [AUROC]: 0.613 [95% CI, 0.550-0.680]). This analysis is shown in Figure 3.

### Comparison of Research Gap Year Applicants with Non-Research Gap Year Applicants

When comparing RGY with non-RGY applicants to the study institution over the seven-year period, RGY applicants had significantly lower median USMLE Step 1 scores (240 vs 245, *P* < 0.001), were less likely to be AOA (16.3% vs 27.2%, *P* < 0.001), and had significantly more total research activities (29 vs 10, *P* < 0.001). Table 4 details a complete comparison of scores, AOA, and research productively between RGY and non-RGY applicants.

**Table 4:** Comparison of Application Variables Between Research Gap Year Applicants and Nonresearch Gap Year Applicants to the Study Institution From 2014 to 2021

	RGY Applicants	Non-RGY Applicants	*P*
Number	558	4537	—
USMLE step 1 score	240 ± 15	245 ± 13	<0.001
USMLE step 2 CK score	247 ± 14	252 ± 13	<0.001
AOA (%)	91 (16.3)	1236 (27.2)	<0.001
Total research activities	29 ± 27	10 ± 14	<0.001

AOA = alpha omega alpha medical honor society, CK = clinical knowledge, RGY = research gap year, USMLE = US Medical Licensing Examination

Continuous data presented as mean ± SD.

## Discussion

This study identified a substantial increase in the percentage of applicants to the study institution who participated in a research gap over the past seven application cycles. On average, 11% of applicants had done a RGY with the most common time being after their third year of medical school (82.7%). The Northeast and Midwest were the most common geographic regions where applicants participated in a RGY. No associations between match success and timing of a RGY were identified. Most RGY applicants matched into orthopaedic surgery (72.8%), and 19.4% of RGY applicants who matched orthopaedics did so at the same institution they did their RGY. Research productivity, denoted as total number of research activities, was weakly associated with match success for RGY applicants. Many significant differences were noted in effective residency application variables between students who pursued a RGY and those who did not do a RGY. Specifically, applicants who did a RGY had significantly lower USMLE Step 1 scores, USMLE Step 2 CK scores, and were less likely to be AOA.

The hypothesis regarding an increase in the percentage of orthopaedic residency applicants to the study institution over the past seven years was confirmed by the strong, positive Pearson correlation coefficient of 0.945 [95% CI, 0.666-0.992]. A paucity of data explaining why orthopaedic residency applicants might be taking a year out from medical school to pursue RGY opportunities is noted. The findings of this study can be used to surmise several reasons. First, most (82.7%) of the applicants who did a RGY in this study elected to do so after their third year of medical school. At that stage of medical school training, most medical students have taken USMLE Step 1 and know which specialty to which they will apply. Students who perceive that they have a low USMLE Step 1 might be counseled that having a productive RGY could strengthen their residency application. The results of this study demonstrated that applicants who did a RGY had, on average, a 5-point lower USMLE Step 1 score than non-RGY applicants. In addition, RGY applicants were less likely to be AOA (16.3% vs 27.2%). Similar findings were reported within otolaryngology-head and neck surgery residency applicants. A recent study reported that 16% of otolaryngology—head and neck surgery applicants from 2014 to 2015 to 2019 to 2020 participated in a RGY and were significantly less likely to be AOA (7.7% vs 92.3%) and had lower mean USMLE Step 1 scores than non-RGY applicants. Of note, the authors reported markedly greater odds of matching into a top-25 ranked otolaryngology program for applicants who did a RGY.^12^ Another reason medical students may seek a RGY is deciding late on a career in orthopaedics. Applicants might pursue a RGY to learn more about the field, cultivate research interests, and identify mentors. Finally, a common reason for pursuing a RGY is not matching into orthopaedics after applying. Kheir et al^13^ conducted a survey study of medical students who applied to orthopaedic residency programs from 2016 to 2019 immediately after match day. The authors compiled 934 responses, of which 81 were identified as not having matched into orthopaedics. Of those 81 applicants, 47 (58.0%) matched into an orthopaedic residency program on either their second (N = 43) or third attempt (N = 3), and one applicant matched after more than five attempts. Interestingly, 48 of those applicants pursued a RGY and had a subsequent match rate of 52.1% compared with a 64.0% chance of matching in the 25 applicants who opted for a general surgery preliminary year.^13^ The findings of this study found greater match success (n = 30/41, 73.1%) for applicants who pursued a RGY after having not matched on the first attempt.

To date, the sole orthopaedic study investigating how RGY might affect match success was a review of a single, large academic institution's 18-year experience with offering RGY opportunities. Egol et al^2^ noted a higher match rate in these students compared with NRMP published data. The authors surveyed 129 students who spent a full-year within the Department of Orthopedic Surgery at their center after college graduation, during or after medical school, or after an unsuccessful first match attempt into orthopaedics. Of the 103 students who completed all questionnaires, 91% successfully matched into an orthopaedics residency, well above the 67.9% average from 2006 to 2018. Furthermore, this match rate was higher despite a four-point lower, on average, USMLE Step 1 score, than publicly published data. The authors noted that the demographics of students participating in a RGY were more frequently women and minorities than the demographics of practicing orthopaedic surgeons.^2^ The results of this study corroborate their findings because RGY applicants had a 72.8% match success in this study, which is slightly higher than the 67.2% match percentage of applicants in the 2020 to 2021 application cycle.^14^ Among all applicants who pursued a RGY, variables associated with match success included a higher median USMLE Step 1 score (243 vs 236 for unmatched RGY applicants), higher USMLE Step 2 CK scores (250 vs 241 for unmatched RGY applicants), AOA (20.3% vs 4.9% for unmatched RGY applicants), and a median five more research activities than unmatched RGY applicants. Of note, the timing of when applicants did a RGY did not affect match success. With USMLE Step 1 moving to pass/fail as early as January 2022, whether research gap year experiences will become more common and more effective in the resident selection process is unknown.^15^

Although RGY applicants had a median 19 more total research activities than non-RGY applicants in this study, the number of research activities were not found to be highly associated with match success (ROC curve cutoff threshold: ≥ 17 total research activities; AUROC: 0.613 [95% CI, 0.550-0.680]). This finding suggests that other aspects of RGY experiences might add value to a student's residency application considering that RGY applicants to our institution had approximately a 5% greater match percentage compared with 2020 to 2021 NRMP data. RGY experiences frequently lead to close working relationships between the student and current orthopaedic residents, fellows, and faculty mentors. In a time where it is becoming increasingly difficult to separate applicants based on multiple factors including USMLE scores, standardized letters of recommendation (LORs) that are glowing in nearly all applicants,^16^ and the fact that many medical schools are not holding AOA elections until after residency applications are submitted, personal connections between current residents, fellows, and faculty can be immensely important. A RGY may afford students a unique, longitudinal opportunity to interact with faculty. A positive RGY could result in strong LORs or personal communications by those faculty with residency programs on a student's behalf. This statement is supported by the nearly 20% of RGY applicants in this study who matched into an orthopaedic residency program at the same institution where they did their RGY. Although the findings of this study provide data on an important topic with limited existing literature, further investigation on a national level, including surveying RGY applicants, is needed to further elucidate why students are pursuing these opportunities and how RGY affects match success into an orthopaedic residency.

Several limitations are noted in this study. This study contained data only from applicants to a single, academic residency program and may not reflect trends at other institutions or over different timeframes. Including international students applying to orthopaedic surgery residency programs in the United States, 7446 total applicants were recorded from 2016 to 2020.^1^ During that period, 4,423 of those applicants (59.4%) applied to the study institution. The rationale for why applicants pursued a RGY was not clear in all circumstances. Comprehensive Google searches were used to try and identify which program applicants matched into; however, not all applicants were successfully identified by this method, which may affect the analysis comparing characteristics between RGY applicants who matched into orthopaedics and those who did not. A method of crossmatching applicants identified by Google search based on year of residency with year of application and matching medical school listed on a residency program site with that of their ERAS application was used. This methodology relies on programs having up to date residency websites, available published abstracts with affiliations listed, or applicants having update professional networking accounts that allow for accurate identification and cross referencing of training background. Although unlikely, possibly, applicants were misidentified by this method. Furthermore, data regarding where applicants did away rotations were not included in ERAS applications and, therefore, were not available for analysis in this study.

## Conclusions

The percentage of RGY applicants to the study institution nearly doubled between 2014 to 15 and 2020 to 21. Most students completing a RGY did so after their third year of medical school. RGY applicants had a higher match rate than nationally published match rates. Applicants who did a RGY had significantly lower USMLE Step 1 scores, USMLE Step 2 CK scores, and were less likely to be AOA. Further study is needed on a national level.
